# Characterization of the Molecular Interplay between *Moraxella catarrhalis* and Human Respiratory Tract Epithelial Cells

**DOI:** 10.1371/journal.pone.0072193

**Published:** 2013-08-06

**Authors:** Stefan P. W. de Vries, Marc J. Eleveld, Peter W. M. Hermans, Hester J. Bootsma

**Affiliations:** Laboratory of Pediatric Infectious Diseases, Radboud University Medical Centre, Nijmegen, The Netherlands; University of Iowa Carver College of Medicine, United States of America

## Abstract

*Moraxella catarrhalis* is a mucosal pathogen that causes childhood otitis media and exacerbations of chronic obstructive pulmonary disease in adults. During the course of infection, *M. catarrhalis* needs to adhere to epithelial cells of different host niches such as the nasopharynx and lungs, and consequently, efficient adhesion to epithelial cells is considered an important virulence trait of *M. catarrhalis*. By using Tn-seq, a genome-wide negative selection screenings technology, we identified 15 genes potentially required for adherence of *M. catarrhalis* BBH18 to pharyngeal epithelial Detroit 562 and lung epithelial A549 cells. Validation with directed deletion mutants confirmed the importance of *aroA* (3-phosphoshikimate 1-carboxyvinyl-transferase), *ecnAB* (entericidin EcnAB), *lgt1* (glucosyltransferase), and MCR_1483 (outer membrane lipoprotein) for cellular adherence, with ΔMCR_1483 being most severely attenuated in adherence to both cell lines. Expression profiling of *M. catarrhalis* BBH18 during adherence to Detroit 562 cells showed increased expression of 34 genes in cell-attached versus planktonic bacteria, among which ABC transporters for molybdate and sulfate, while reduced expression of 16 genes was observed. Notably, neither the newly identified genes affecting adhesion nor known adhesion genes were differentially expressed during adhesion, but appeared to be constitutively expressed at a high level. Profiling of the transcriptional response of Detroit 562 cells upon adherence of *M. catarrhalis* BBH18 showed induction of a panel of pro-inflammatory genes as well as genes involved in the prevention of damage of the epithelial barrier. In conclusion, this study provides new insight into the molecular interplay between *M. catarrhalis* and host epithelial cells during the process of adherence.

## Introduction

*Moraxella catarrhalis* is a human-restricted pathogen that is responsible for respiratory tract infections such as childhood otitis media (OM) and exacerbations of chronic obstructive pulmonary disease (COPD) in adults [1]. Successful colonization and infection by *M. catarrhalis* depends on its ability to attach to the respiratory tract mucosa. Various molecular typing methods, including multi-locus sequencing-typing, have demonstrated the existence of two phylogenetic lineages within the *M. catarrhalis* species. Isolates grouped into the more virulent lineage 1 are more frequently isolated from diseased individuals and adhere more efficiently to respiratory tract epithelial cells than do isolates of lineage 2 [2,3].

Adhesion is a multifactorial process mediated by many adhesin molecules including fimbrial adhesins such as type IV pili [4] and non-fimbrial adhesins like the Ubiquitous surface proteins A1 and A2H (UspA1 and UspA2H) [5], the *M. catarrhalis* IgD-binding protein/haemagglutinin (MID/Hag) [6], outer membrane protein CD (OMP CD) [7], and *M. catarrhalis* adhesion protein (McaP) [5], recently reviewed by Su et al. [8],. Of importance, the UspA proteins are built out of interchangeable sequence motifs and consequently large variation exists between isolates, which could affect the adherence capability of each isolate [9]. Strain to strain variation in adhesion efficiency is also reported to be dependent on adhesin expression as isolates with low MID/Hag [6] or UspA1 [10,11] expression showed reduced adhesion. The different adhesins bind to a variety of receptors or structural molecules expressed on respiratory tract epithelial cells: UspA1 for instance mediates adhesion through binding to carcinoembryonic antigen-related cellular adhesion molecule 1 (CEACAM1) [12], and to the ECM proteins fibronectin [13] and laminin [14]. Host receptors for other adhesin molecules of *M. catarrhalis* such as MID/Hag or McaP remain to be identified. Further, Ahmed et al. postulated that the negative charge of *M. catarrhalis* may be important for binding to positively charged surface structures present on pharyngeal epithelial cells [15]. The complete repertoire of adhesion mechanisms is proposed to allow *M. catarrhalis* to attach to epithelial cell types of different anatomical niches [8].

The interaction of *M. catarrhalis* and its human host is a dynamic process and imbalance or failure to induce a proper innate immune defense is thought to enable *M. catarrhalis* to expand and persist in the human airways [16]. Transcriptional reprogramming of respiratory tract epithelial cells upon contact with *M. catarrhalis* is considered to be central to the host defense. The upper airway epithelial cells play a key role together with macrophages, dendritic cells, neutrophils, and mast cells in steering the host inflammatory response against *M. catarrhalis*. This inflammatory response is trigged by binding of pathogen-associated molecular patterns (PAMPs) such as the *M. catarrhalis* lipooligosaccharide [17], to pattern-recognition receptors (PRRs). The resulting activation of signal transduction pathways [18] by *M. catarrhalis* is mainly dependent on Toll-like receptor (TLR)-2 and drives NF-κB-mediated production of interleukin-8 (IL-8), which guides granulocyte recruitment to the site of infection [18,19]. Increased secretion of the pro-inflammatory cytokines IL-6, IL-1β, and IL-8 and granulocyte-macrophage colony-stimulating factor (GM-CSF/CSF3) by airway epithelial cells and macrophages is characteristic of *M. catarrhalis* infections [8]. Interestingly, through UspA1 interaction with CEACAM1 on epithelial cells *M. catarrhalis* is able to partially suppress IL-8 production [20].

The aim of this study was to increase our understanding of the complex interaction of *M. catarrhalis* and its human host during the first critical strep of infection, adherence to epithelial cells. Although several factors are already known to facilitate *M. catarrhalis* adherence, the complete repertoire of adhesins and genes indirectly affecting adhesion has not been completely characterized yet. Here, we have used the genome-wide negative selection screenings technology Tn-seq to identify novel bacterial factors influencing adhesion of *M. catarrhalis* to pharyngeal Detroit 562 and lung A549 epithelial cell lines. The findings of our transposon mutant library screen were validated by testing a panel of directed gene deletion mutants for their ability to adhere. To gain insight into the multifactorial interaction that takes place between the pathogen and the host, the transcriptional responses of both were monitored during adherence of *M. catarrhalis* to Detroit 562 cells.

## Results and Discussion

### Identification of genes affecting *M. catarrhalis* BBH18 adherence

To comprehensively identify the genes relevant for cellular adherence of *M. catarrhalis*, a genome-wide negative selection Tn-seq screen [21,22] was performed. For this a ~7,000 *mariner* transposon mutant library was generated in *M. catarrhalis* BBH18 as described previously [23], and screened during adherence to two respiratory tract epithelial cells of distinct anatomical locations, the pharynx (Detroit 562 cells) and lung (A549 type-II alveolar epithelial cells). Examination of the relative abundance of each individual transposon mutant in the library under control conditions (not exposed to epithelial cells) by Tn-seq revealed transposon insertions covered by at least 10 sequence reads in 5,543 unique TA-sites, representing a total of 1,077 genes.

**Figure 1:**
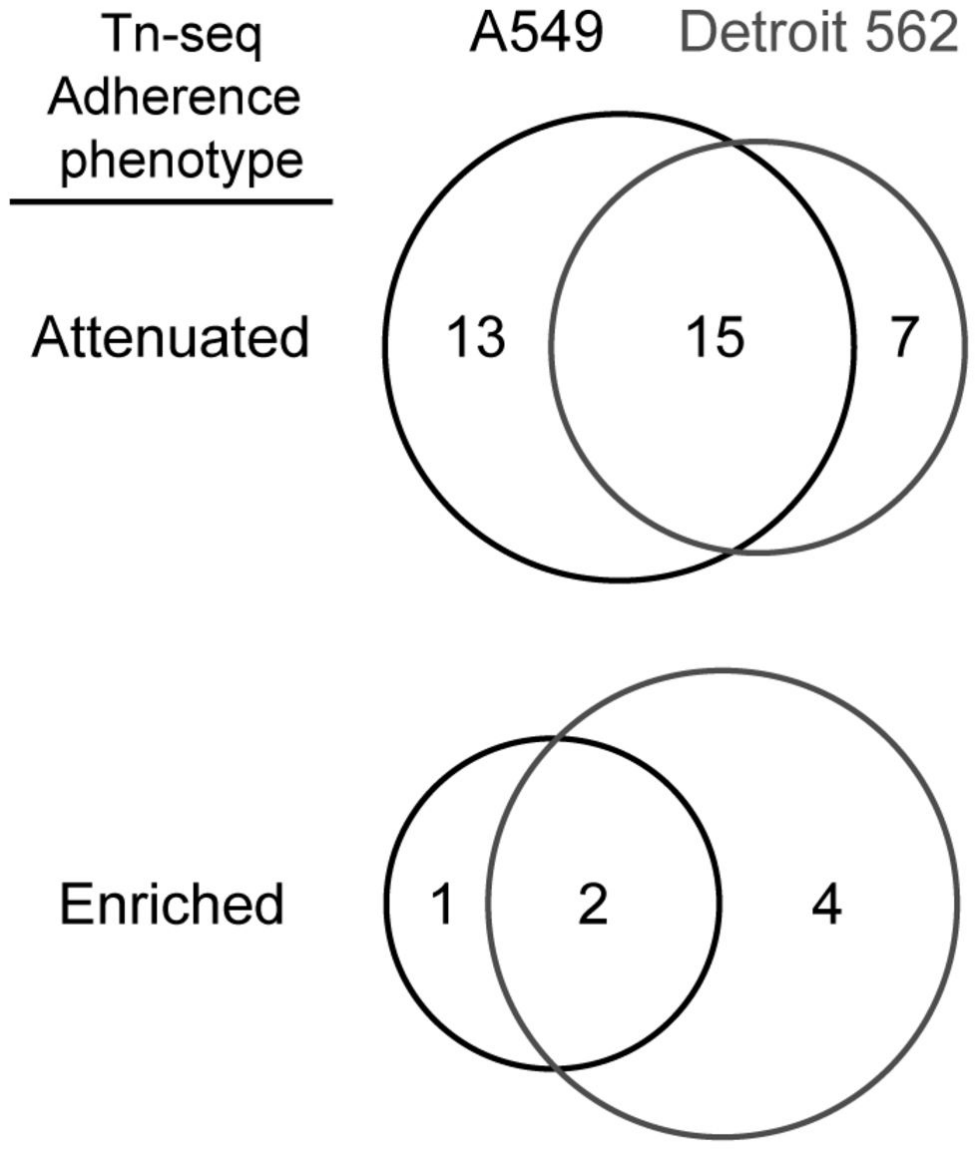
Genes affecting adherence of *M. catarrhalis* to respiratory tract epithelial cells. Overlap between genes identified by Tn-seq as important for adherence of *M. catarrhalis* BBH18 to A549 and Detroit 562 epithelial cells.

For the adherence Tn-seq screen, the mutant library was allowed to attach to A549 and Detroit 562 cells for 1 hour. Subsequently, adherent, non-adherent, and control (incubation in the infection medium alone) fractions were recovered and the relative abundance of each transposon mutant in all fractions was profiled using Tn-seq. Genes of which transposon mutants were attenuated in adherence were identified by a significant fold-change of at least log_2_ 1.6 between the adherent and non-adherent library fractions. To accommodate for potential effects, either positive or negative, of epithelial cells on the composition of the non-adherent fraction, we also included a minimum log_2_ fold-change of 1.2 between the adherent fraction and the library that was incubated in the infection medium alone (control). Based on these selection criteria, 35 genes were found to be required for cellular adherence of BBH18, of which 15 were identified with both Detroit 562 and A549 cells, 13 were specific for A549, and 7 were specific for Detroit 562 (Figure 1 and Table S1). Conversely, insertional inactivation of seven genes appeared to result in enhanced cellular adherence: two for both cell lines, one specific for A549, and four for Detroit 562 (Figure 1 and Table S1).

Of the 35 Tn-seq-identified genes potentially required for *M. catarrhalis* BBH18 adherence, 21 genes are classified as (conserved) hypothetical or of unknown function. Four genes belong to the functional class of “cell envelop”, namely *uspA2H*, *lgt1* involved in LOS biosynthesis [24], the putative lipoprotein MCR_1483, and the outer membrane protein MCR_1742. Further, genes were identified that are involved in DNA repair (*ruvA* and the putative holiday junction resolvase MCR_1850), cellular processes (entericidin *ecnAB*), energy metabolism (*pfkB* family carbohydrate kinase MCR_1654 and adenosyl-homocysteinase *ahcY*), biosynthesis of aromatic amino acids (*aroA*), regulatory functions (*badM/rrf2* MCR_0609 and *metR* family transcriptional regulator MCR_0330), transcription (endoribonuclease L-PSP MCR_0348), and protein synthesis (*trmB*) (Table S1).

Our screen did not identify all genes previously shown to be involved in cellular adhesion of *M. catarrhalis* such as genes encoding UspA1 [5], MID/Hag [6], OMP CD [7], and McaP [5]. Some of these, such as *ompCD*, were not represented by mutants in the library, likely because *ompCD* mutants have attenuated *in vitro* growth [7]. Furthermore, we most likely haven’t reached saturation for the number of genes that could potentially be hit by a transposon with our mutant library. However, we deliberately constructed a smaller library to ensure sufficient coverage of each mutant in the fractions samples (at least 200-fold), and thus prevent random loss of mutants from the library during the screen (i.e., false-positives).

Another possible factor limiting the number of genes identified in our screen may be the relatively small level of attenuation of single mutants. For instance, we have previously shown that a BBH18 mutant of *uspA1*, one of the main adhesion factors, still adhered to Detroit 562 and A549 cells at levels 34% and 57% of wild-type, respectively [23]. This could possibly originate in outer membrane surface charge mediating non-specific adhesion [15] and/or the fact that *M. catarrhalis* adhesion is a multifactorial process. The complex interplay between multiple adhesin molecules was also demonstrated by Bullard et al. who showed that while purified recombinant MID/Hag bound to A549 cells, expression of MID/Hag in *Escherichia coli* did not enhance binding to A549 cells, whereas binding of MID/Hag-expressing *E. coli* to human middle ear epithelial cells (HMEE) was increased [25]. It was proposed that the MID/Hag interaction with A549 requires co-expression of UspA1 and/or other adhesins such as OMP CD [25]. As a consequence, loss of a single adhesin does not completely abolish adhesion, also exemplified by Timpe et al. who demonstrated that reduced adherence after deletion of *mcaP* from strain O35E was only observed in a *uspA1, uspA2*, and *mid/hag* triple mutant background and not in wild-type [26]. Along this line, screening a transposon mutant library generated in an *uspA1* mutant background may result in the identification of more genes affecting adhesion.

### Adherence efficiency of directed gene deletion mutants

**Figure 2:**
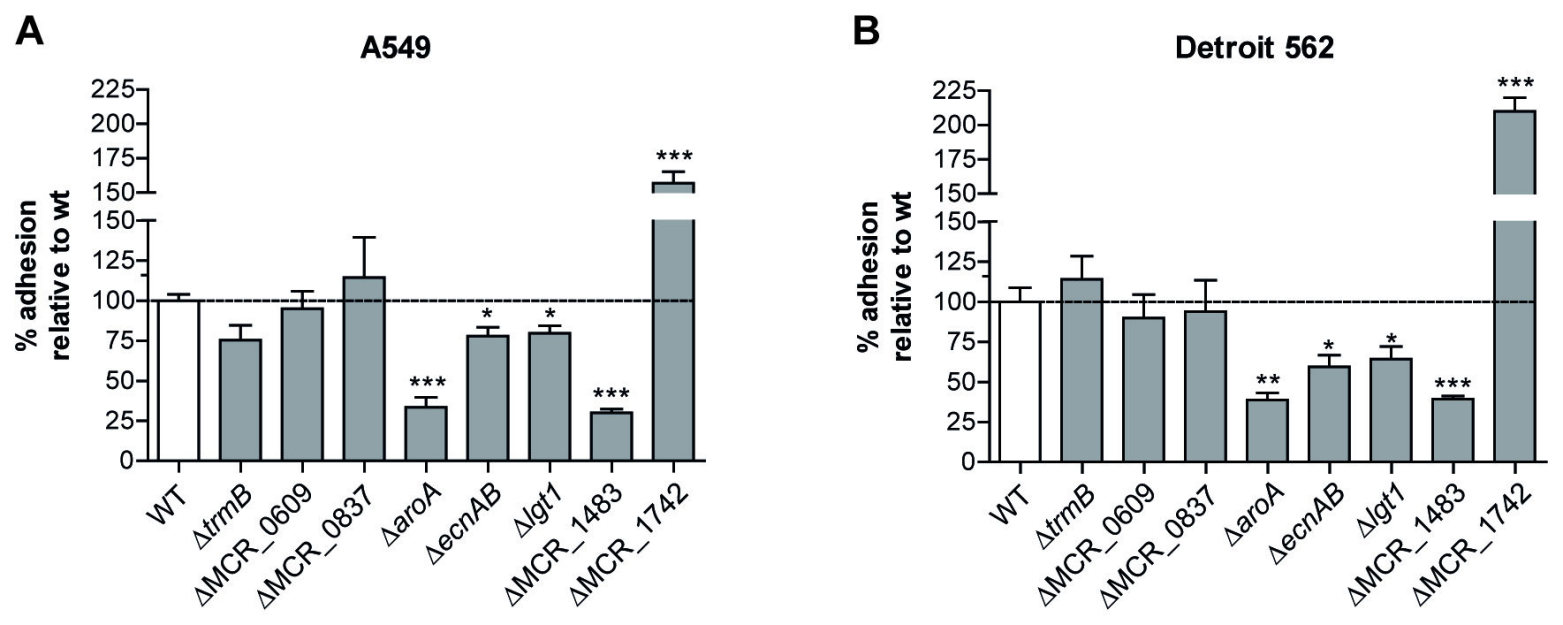
Adhesion of directed gene deletion mutants to respiratory tract epithelial cells. Adherence of *aroA, ecnAB*, lgt1, and the putative lipoprotein MCR_1483 to both Detroit 562 (A) and A549 (B) cells was significantly attenuated compared to wild-type (WT). Adherence levels are expressed relative to WT (n ≥ 3) and shown as means and SEM. Statistical difference was determined with a Mann-Whitney test with * *P* < 0.05, ** *P* < 0.01, and *** *P* < 0.001.

Since the focus of this study was to identify novel factors that affect adhesion of *M. catarrhalis*, we generated directed gene deletion mutants for a selection of eight genes (MCR_0609, MCR_0837, *aroA*, *ecnAB*, *lgt1*, MCR_1483, and MCR_1742) that have not previously been linked to adhesion (Table 1). Importantly, deletion of these genes did not affect growth or survival of BBH18 in the infection medium during 1 hour (data not shown). Adherence of mutants of MCR_1483, *aroA, lgt1*, and *ecnAB* to both A549 and Detroit 562 cells was significantly attenuated compared to wild-type, confirming the Tn-seq results (Figure 2AB). Mutants of *trmB*, MCR_0609, and MCR_0837, however, displayed wild-type adherence levels, while deletion of MCR_1742, encoding an uncharacterized outer membrane protein, even appeared to result in enhanced adhesion to both cell lines (Figure 2AB). This apparent discrepancy may be explained by the difference in experimental setup between the Tn-seq screen and the validation experiments: adherence of transposon mutants during the screen was assessed in a competition setup (i.e., between a particular mutant and the remainder of the library), whereas directed deletion mutants were assayed individually for their adherence ability in validation experiments, which is a less stringent approach as has also been shown by others [27,28]. Furthermore, differences in inter-bacterial interaction dynamics may have resulted in the increased adherence of the MCR_1742 deletion mutant.

**Table 1:** Selection of Tn-seq identified genes required for adherence of *M. catarrhalis* BBH18.

				Tn-seq log_2_ adherent / non-adherent	
Locus tag	Gene	Product	Functional class	A549	Detroit
MCR_0888	*aroA*	3-phosphoshikimate 1-carboxyvinyltransferase	Amino acid biosynthesis	-2.7	-1.8
MCR_1095	*lgt1*	glucosyltransferase Lgt1	Cell envelope	-3.3	-2.6
MCR_1483	-	putative lipoprotein	Cell envelope	-2.3	-1.7
MCR_1742	-	outer membrane protein	Cell envelope		-2.4
MCR_1029	*ecnAB*	entericidin EcnAB	Cellular processes	-2.3	-1.9
MCR_0343	*trmB*	tRNA (guanine-N(7)-)-methyltransferase	Protein synthesis	-3.2	-2
MCR_0609	-	BadM/Rrf2 family transcriptional regulator	Regulatory functions	-3.8	
MCR_0837	-	putative phosphohistidine phosphatase	Unknown function	-2.9	-2.2

Loss of the putative outer membrane lipoprotein gene MCR_1483 and *aroA* resulted in the most severe attenuation of adherence, to 30-39% and 34-39% of wild-type, respectively. MCR_1483 encodes a small 48 amino acid putative lipoprotein sharing 58% identity with the LppL protein of *Pseudomonas* *sp.* GM102. In *M. catarrhalis* BBH18 MCR_1483 is part of a putative operon together with *lysA, dapF*, and *xerC*, whereas the representative gene cluster in *Pseudomonas* *sp.* contains two additional uncharacterized ORFs [29]. In *Pseudomonas fluorescens* WCS365 this operon, more specifically *xerC* encoding a site specific recombinase, is required for root colonization [30]. Others postulated that in *Pseudomonas* *sp*. LppL is involved in regulation of the lysine biosynthesis gene *lysA* [29]. The attenuated adhesion of the *aroA* mutant is intriguing, especially since a direct role of this predicted cytoplasmic protein in cellular adhesion is unlikely. The *aroA* gene encodes a 3-phosphoshikimate 1-carboxyvinyltransferase that is involved in aromatic amino acid metabolism and generation of chorismate-derived metabolites [31]. Deletion of *aro* genes leads to attenuated virulence in many bacterial pathogens and often results in defective cell envelope biosynthesis [32]. It could be possible that a defective outer membrane function in *M. catarrhalis* BBH18Δ*aroA* caused in incorrect localization of “true” adhesins, thereby leading to attenuated adhesion. Interestingly, both MCR_1483 and *aroA* were also found to be required for complement resistance of *M. catarrhalis* (De Vries et al., unpublished data) and thus appear to fulfill or affect an essential virulence mechanism. More subtle effects on adherence efficiency were observed upon deletion of *ecnAB* and *lgt1*, 59-78% and 64-80% of wild-type, respectively. In *E. coli, ecnAB* is reported to direct the biosynthesis of two small outer membrane lipoproteins that regulate programmed cell death [33]. Deletion of *lgt1*, which encodes an α-(1,2) glucosyltransferase involved in LOS biosynthesis [24], is expected to result in a truncated LOS structure. An altered LOS structure may lead to decreased membrane stability and altered surface display of adhesins as has been shown for a LOS-deficient mutant of strain O35E [34].

### Differential expression of *M. catarrhalis* upon adhesion

To monitor changes in the transcriptional profile of *M. catarrhalis* BBH18 early after adherence to upper airway epithelial cells, we compared expression of the adherent (cell-attached) and non-adherent (planktonic) fractions after 1h adherence to Detroit 562 cells. In addition, we examined expression of bacteria that were only incubated in the infection medium (control fraction). Rather surprisingly, very few expression differences (~3-fold increased expression of *lbpB* and MCR_0218) were observed between control and non-adherent bacteria, indicating that in the absence of direct contact, host cells had a limited impact on *M. catarrhalis*, and that the non-adherent fraction is likely to represent basal expression levels under our assay conditions. Therefore, we focused our analysis on the differences between the cell-attached and non-adherent fractions.

**Figure 3:**
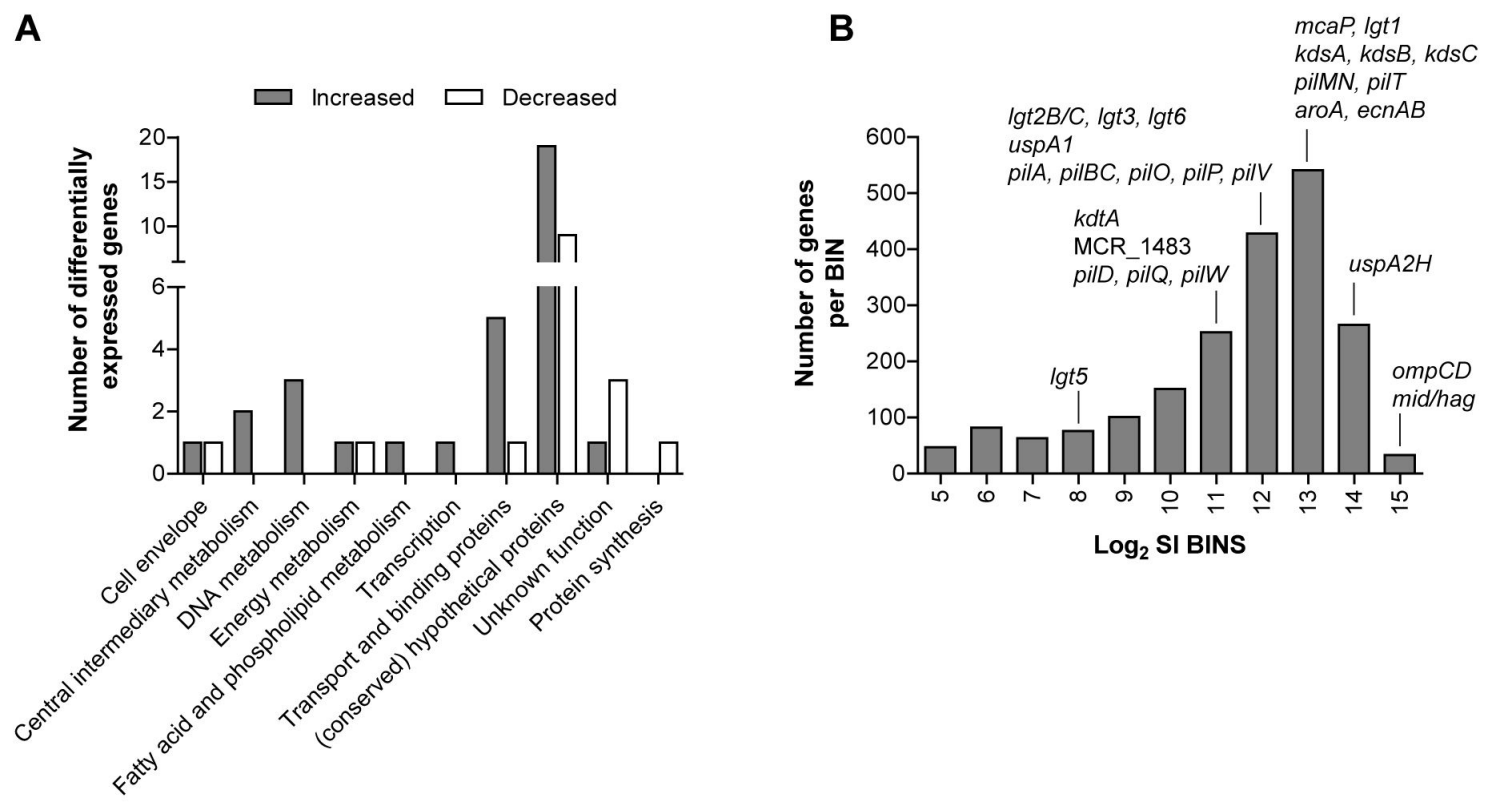
Gene expression of *M. catarrhalis* BBH18 during adherence to Detroit 562 cells. A) Functional class distribution of genes differentially expressed in cell-attached relative to non-adherent (planktonic) *M. catarrhalis* (n = 4), depicted as number of genes per functional class category. B) Distribution of gene expression levels of known adhesins and structural components associated with adherence in cell-attached *M. catarrhalis*. Average log_2_ microarrays signal intensities (SI) per gene were grouped into bins (1-log interval per bin), and the total number of genes within a bin is shown here.

In total, we found increased expression of 34 genes and reduced expression of 16 genes in adherent relative to non-adherent bacteria (Table S2). Next to (conserved) hypothetical genes (19, 55.8%), predominant classes found among the genes with increased expression in the cell-attached fraction were metabolic genes (7, 20.6%) and genes encoding “transport and binding” proteins (5, 14.7%) (Figure 3A). For example, increased expression was found for the putative acyl-CoA dehydrogenase gene *fadE* (MCR_0042) involved in beta-oxidation of fatty acids [35], and for ABC transporter genes for molybdate (*modABC-*cluster). An increased need for molybdenum could arise due to higher redox activity in sulfur, nitrogen en carbon metabolism for which molybdenum is required [36]. In line with this, gene expression of nitrate reductase (*narJ*), encoding a molybdenum cofactor-dependent enzyme [36], was also increased, potentially to fulfill the energy need required for adaptation during epithelial cell adherence. In addition, several sulfate metabolism genes displayed increased expression in cell-attached bacteria, namely ABC transporter genes that mediate uptake of sulfur (*cysP* and *cysU*) and genes that participate in biosynthesis of reduced sulfur metabolites (*cysH* and *cysI*). Interestingly, deletion of *cysH* from *Mycobacterium tuberculosis* resulted in attenuated virulence in mice and reduced resistance against oxidative stress [37]. The majority of lower expressed genes in cell-attached bacteria were (conserved) hypotheticals (9, 56.3%), but reduced expression was also found for genes encoding a putative lipoprotein (MCR_1168), glutaredoxin-like protein (MCR_0600), and 50S ribosomal protein L33 (*rpmG*).

Interestingly, none of the genes encoding the known *M. catarrhalis* adhesion factors UspA1/A2H [5], MID/Hag [6], OMP CD [7], McaP [5], and LOS [38] and type IV pili biosynthesis genes [4] were differentially expressed in cell-attached bacteria. Grouping of the microarray log_2_ signal intensities obtained for each gene in cell-attached bacteria into bins (1-log intervals) showed that the adhesin genes *ompCD, mid/hag*, and *uspA2H* were among the highest expressed genes (Figure 3B). This may suggest that they are constitutively expressed at relatively high level, and are not subject to transcriptional regulation on a population level, although transcriptional and translational phase variation has been shown to affect expression of UspA1/A2H [39] and MID/Hag [40] at single bacterium level.

### Differential host gene expression upon *M. catarrhalis* adhesion

Next to the bacterial response upon epithelial cell adhesion, we examined the concurrent host response during attachment of *M. catarrhalis* to Detroit 562 cells. After 2h adherence of *M. catarrhalis* BBH18, 77 Detroit 562 genes showed a more than 2-fold enhanced expression relative to control Detroit 562 cells incubated in the infection medium alone, whereas reduced expression was only observed for thioredoxin-interacting protein TXNIP (Table S3). In particular, expression of various pro-inflammatory genes was increased during attachment of *M. catarrhalis* (Table 2), namely of genes encoding tumor necrosis factor (TNF), Interleukin (IL)-1β, IL-6, IL-8, IL-17C, colony stimulating factor 2 and 3 (G-CSF and GM-CSF, respectively), and chemokines CXCL1, CXCL2, CXCL3, and CCL20. These genes are known to be induced during acute infection by many bacterial pathogens, and serve as a general alarm signal that increases the likelihood that the infection is detected by the host [41]. The increased expression (~129-fold) of CCL20 was most pronounced. CCL20 is a strong attractant for lymphocytes and weakly chemotactic for granulocytes [42], and can also be induced by IL-1β [43]. Epithelial cells are the main source of IL-17C and its receptor (IL-17RE/IL-17RA heterodimer) is predominantly expressed on epithelial cells. Therefore, the increased expression of IL-17C is expected to result in autocrine signaling to enhance gene expression of factors that regulate the local and systemic response [44]. In line with other studies, binding of *M. catarrhalis* to Detroit 562 cells induced expression of the cell adhesion molecule ICAM1, which regulates leukocyte recruitment and facilitates neutrophil bacterial killing [17].

**Table 2:** Selection of Detroit 562 genes induced in response to *M. catarrhalis* BBH18.

Gene	Description	Log_2_ FC^a^	
*Immune system*			
CCL20	chemokine (C-C motif) ligand 20	7.0	
IL1A	interleukin 1, alpha	5.3	
CD69	CD69 molecule	5.2	
CXCL3	chemokine (C-X-C motif) ligand 3	5.0	
IL1Β	interleukin 1, beta	4.8	
TNF	tumor necrosis factor (TNF superfamily, member 2)	4.2	
IL8	interleukin 8	4.1	
SERPINA3	serpin peptidase inhibitor, clade A (alpha-1 antiproteinase, antitrypsin), member 3	4.0	
IL6	interleukin 6	4.0	
PTX3	pentraxin-related gene, rapidly induced by IL-1beta	4.0	
IL29	interleukin 29	3.6	
CSF3 (G-CSF)	colony stimulating factor 3 (granulocyte)	3.5	
NFKBIZ	nuclear factor of κ light polypeptide gene enhancer in B-cells inhibitor, zeta	3.2	
TA-NFKBH	T-cell activation NFκB-like protein	3.2	
JUN	v-jun sarcoma virus 17 oncogene homolog (avian)	3.1	
LTB	lymphotoxin beta (TNF superfamily, member 3)	3.0	
IL17C	interleukin 17C	2.7	
IL1F9	interleukin 1 family, member 9	2.6	
ICAM1	intercellular adhesion molecule 1 (CD54), human rhinovirus receptor	2.5	
IRAK2	interleukin-1 receptor-associated kinase 2	2.3	
CSF2 (GM-CSF)	colony stimulating factor 2 (granulocyte-macrophage)	2.2	
CXCR4	chemokine (C-X-C motif) receptor 4	2.2	
EDN 2	endothelin 2	2.1	
IL32	interleukin 32	2.1	
TNFSF18	tumor necrosis factor (ligand) superfamily, member 18	2.1	
MAP3K8	mitogen-activated protein kinase kinase kinase 8	2.1	
LIF	leukemia inhibitory factor	2.0	
CXCL1	chemokine (C-X-C motif) ligand 1	1.9	
CXCL2	chemokine (C-X-C motif) ligand 2	1.9	
NFKBIA	nuclear factor of kappa light polypeptide gene enhancer in B-cells inhibitor, α	1.9	
TIFA	TRAF-interacting protein with a forkhead-associated domain	1.6	
ICOSLG	inducible T-cell co-stimulator ligand	1.5	
EDN 1	endothelin 1	1.4	
CD83	CD83 molecule	1.3	
ATF3	activating transcription factor 3	1.2	
*Apoptosis and negative regulation of apoptosis*			
ANGPTL4	angiopoietin-like 4	4.4	
TNFAIP3	tumor necrosis factor, alpha-induced protein 3	4.0	
C8orf4	chromosome 8 open reading frame 4	3.3	
BIRC3	baculoviral IAP repeat-containing 3	3.1	
ZC3H12A	zinc finger CCCH-type containing 12A	2.8	
DDIT4	DNA-damage-inducible transcript 4	1.1	
BCL2A1	BCL2-related protein A1	1.7	
IER3	immediate early response 3	1.3	

a FC; fold-change BBH18-infected/mock-infected Detroit

Enhanced gene expression was observed for various transcriptional regulators and components of signal transduction pathways such as the transcription activator JUN, and the cyclic AMP-dependent transcription factor ATF-3. In addition, increased expression of immune dampening factors was found for the NF-κB pathway suppressors IκB-α (NFKBIA), acting in the cytoplasm to sequester NF-κB, and the kinase MAP3K8 [41]. The panel of immune suppressors was extended by increased expression of TNFAIP3, which functions in a negative feedback loop regulating TLR-ligand and TNF induced responses [45].

Noteworthy, *M. catarrhalis* binding induced expression of α-1-antichymotrypsin (SERPINA3), a serine protease inhibitor that protects host tissue from oxidative and proteolytic damage [46] in order to counteract the action of neutrophil secreted factors such as elastase, metalloproteases and reactive oxidative radicals. Previously it was proposed that UspA-mediated α-1-antichymotrypsin binding by *M. catarrhalis* confers resistance against host proteases. This potentially allows *M. catarrhalis* to induce more severe inflammation, which may result in excessive tissue damage and subsequently exposure of ECM that facilitates bacterial attachment and colonization [8,46]. Of note, upon binding of *M. catarrhalis* expression of anti-apoptotic genes BCL2A1 and BIRC3 was also increased, potentially compensating for apoptotic stimuli and preventing damage to the epithelial barrier (Table 2).

Overall, the abundantly induced inflammatory response characterizing *M. catarrhalis* airway inflammation is key to infiltration of neutrophils, lymphocytes, and macrophages, and contributes to COPD exacerbation and OM pathogenesis [8,47]. The potential of *M. catarrhalis* to induce high levels of inflammation may cause higher disease burden during co-infection with other respiratory tract pathogens such as *S. pneumoniae* and NTHi. For *S. pneumoniae*, a pro-inflammatory environment has been shown to be required for progression to the middle ear and its ability to cause OM, exemplified by Short et al. who demonstrated that viral or lipopolysaccharide-induced inflammation is sufficient for *S. pneumoniae* to cause OM in an infant murine model [48]. Interestingly, co-infection of *M. catarrhalis* with *S. pneumoniae* resulted in increased incidence of OM, higher pneumococcal load, and prolonged infection in a murine nasopharyngeal colonization model [49].

## Conclusions

In this paper, we used a combination of genome-wide approaches to increase our understanding of the molecular interplay between *M. catarrhalis* and respiratory tract epithelial cells during cellular adhesion, which is the critical first step towards colonization and infection. One of the newly identified factors that affect *M. catarrhalis* adhesion was the putative outer membrane lipoprotein MCR_1483. Both host and microbe were found to undergo transcriptional adaptation during adherence, with expression changes in cell-attached *M. catarrhalis* mainly restricted to metabolism and transport. In the epithelial cells, a significant induction of a pro-inflammatory response was observed, which may affect disease progression during (poly)-microbial respiratory tract infections. The results described in this study should provide basis for future investigations characterizing *M. catarrhalis* pathogenesis.

## Materials and Methods

### Bacterial strains and growth conditions

All strains used in this study are listed in Table S4. *M. catarrhalis* was cultured on brain heart infusion (BHI) agar plates at 37° C in an atmosphere containing 5% CO_2_ or in BHI broth at 37° C at 200-250 rpm. *M. catarrhalis* BBH18 transposon mutant libraries and gene deletion mutants were cultured the presence of 30 µg ml^-1^ spectinomycin (BHI-spec). Aliquots of bacteria were routinely stored in the presence of 20% glycerol at -80° C.

### Construction of a *M. catarrhalis* transposon mutant library

A *M. catarrhalis* BBH18 mariner transposon mutant library consisting of ~7,000 independent transformants was generated as described in De Vries et al. [23].

### Generation of *M. catarrhalis* directed gene deletion mutants

Directed gene deletions were introduced in *M. catarrhalis* BBH18 by allelic exchange of the target gene with a spectinomycin resistance cassette as described in De Vries et al. [23]. Chromosomal DNA of a first generation mutant was used to PCR-amplify the spectinomycin resistance cassette and the gene flanking regions using the L1 and R1 primers (Table S5). The obtained PCR product was subsequently introduced into a wild-type recipient by natural transformation. At the same time, competent cells were processed through the transformation procedure without addition of DNA to obtain a coupled wild-type strain. Primers used in this study are shown in Table S5.

### Adhesion assays with respiratory tract epithelial cells

Adhesion assays with the human pharyngeal epithelial cell line Detroit 562 (ATCC CCL-138) and the type II alveolar epithelial cell line A549 (ATCC CCL-185) were performed essentially as described previously [23]. Both cell lines were routinely cultured in DMEM with GlutaMAX™-I and 10% fetal calf serum (FCS) (Invitrogen) at 37° C and 5% CO_2_. For adherence assays, monolayers of ~1·10^6^ (24-well format) or ~4·10^6^ (6-well format) cells per well were infected at a multiplicity of infection (MOI) of 10 bacteria per cell in 1 ml or 4 ml infection medium (DMEM with 1% FCS), respectively. Non-adherent bacteria were removed by 3 washes with PBS, after which 1% saponin (Sigma Aldrich) in PBS supplemented with 0.15% gelatin was added to detach and lyse eukaryotic cells. CFUs were determined by plating 10-fold serial dilutions.

### Identification of genes affecting adherence using Tn-seq

For the Tn-seq screen, the mutant library was first pre-cultured until mid-log phase (OD_620nm_ ~ 1.0). Transposon mutants were allowed to bind to the epithelial cells for 1 h (n = 4) in a 24-well format. Non-adherent bacteria were collected from the supernatant and the adherent bacteria were obtained after lysis of the eukaryotic cells. In addition, the transposon mutant library was incubated in the infection medium without eukaryotic cells (control). Equivalent CFUs of the recovered adherent and non-adherent, and control fractions were expanded to an OD_620nm_ of 0.2-0.3. Of these cultures, 0.4 ml was mixed with 0.6 ml 50% glycerol in BHI medium and stored at -80° C. The obtained aliquots were expanded to OD_620nm_ of 0.2-0.4 and chromosomal DNA was isolated using Genomic-tip 20/G columns. Tn-seq technology was used to profile the relative abundance of each mutant in all fractions, essentially as described in Burghout et al. [21]. For data analysis, FASTQ files were processed via the ESSENTIALS data analysis pipeline (http://bamics2.cmbi.ru.nl/websoftware/essentials/essentials_start.php) [50]. Tn-seq bar code sequences were used to attribute sequence reads to individual samples. Sequence reads were aligned to the BBH18 reference genome [35] with a minimum match of 16 nt and collected per unique insertion site (TA dinucleotide sequence) and per gene. The number of unique transposon insertion sites was determined using the control fraction only samples and defined as the positions that were covered by an average of at least 10 sequence reads. If sequence reads were mapped to both sites of the transposon insertion site, the average sequence reads of the 5’ and 3’ were used. Selection criteria for genes affecting adherence were as follows: (1) a log_2_ fold-change in sequence reads between the adherent and non-adherent fraction smaller than -1.6 or larger than 1.6 and an adjusted *P*-value < 0.05 and (2) a log_2_ fold-change in sequence reads between the adherent and the control fraction < -1.2 or > 1.2. Genes of which transposon mutants were predicted by ESSENTIALS [50] to be essential or have a severely reduced fitness, defined as a log_2_ fold-change between actual sequence reads and calculated expected sequence reads < -3.84, were excluded from our analysis.

### Validation experiments using directed gene deletion mutants

Directed mutants were first pre-cultured until mid-log phase (OD_620nm_ ~ 1.0) and stored at -80° C in the presence of 20% glycerol. Adhesion assays with directed mutants on A549 and Detroit 562 cells were performed in a 24-well format. Mutants were allowed to bind during 1 h (n ≥ 3). The percentage adherence of the directed mutant strains was calculated relative to the percentage adhesion of the wild-type and statistical significance was determined with a Mann-Whitney test in GraphPad Prism 5.0 (GraphPad Software).

### Gene expression profiling of *M. catarrhalis* during adherence

Expression profiling of wild-type *M. catarrhalis* BBH18 during adhesion to Detroit 562 was conducted in a 6-well format. After 1h adherence, non-adherent bacteria of two wells (6-8 ml) were combined and 2 volumes of RNAprotect Bacteria Reagent (Qiagen) was added. Subsequently, wells were washed 3 times with PBS, 1 ml RNAprotect Bacteria Reagent was added to each well, adherent bacteria were harvested by scraping, and pooled per 2 wells. The bacteria were also incubated in the infection medium without eukaryotic cells and after 1h, 8 ml RNAprotect Bacteria Reagent was added to 4 ml bacterial suspension. In addition, uninfected Detroit 562 cells were lysed with 1 ml RNAprotect Bacteria Reagent. After 5 min incubation at room temperature and harvesting by centrifugation, pellets were stored at -80° C. Total RNA was isolated for the adherent fraction (n = 4), non-adherent fraction (n =4), bacteria without Detroit 562 cells (n =3), and uninfected Detroit 562 cells (n = 1), and contaminating genomic DNA was removed by treatment with Turbo DNase (Ambion) as previously described [35]. RNA obtained from the adherent fraction was enriched for bacterial RNA using the MICROBEnrich kit (Ambion) according to manufacturer’s instructions. Thereafter, RNA was used to generate Cy3-labeled cDNA according to standard Nimblegen gene expression array protocols and hybridized to custom-designed Nimblegen *M. catarrhalis* BBH18 4-plex 72K expression arrays, described in De Vries et al. [23]. Gene expression data was normalized using ArrayStar software (DNASTAR) with Quantile RMA normalization. Probes that cross-hybridized with Detroit 562 cDNA, defined by a normalized log_2_ signal intensity (SI) > 8.965, were omitted from the analysis. Statistical analysis was performed in ArrayStar using a moderated t-test corrected with a Benjamini & Hochberg false discovery rate and considered significantly differentially expressed at an adjusted *P* < 0.05 and a log_2_ fold-change of >1 or <-1. Functional class distribution was assessed using the Institute for Genomic Sciences (IGS) classification (15) with the Fishers exact (one-tail) test, corrected for multiple testing according to Storey and Tibshirani [51].

### Gene expression profiling of Detroit 562 pharyngeal epithelial cells during adherence of *M. catarrhalis*

*M. catarrhalis* BBH18 wild-type was allowed to bind to Detroit-562 cells (n = 6) for 2h (6-well format). After removal of the unbound bacteria, Detroit 562 cells were lysed directly in the well by addition of 1 ml RLT buffer (Qiagen) and collected by scraping. As a control (n = 6), Detroit 562 were incubated in the infection medium alone. Total RNA was isolated with the RNeasy mini kit (Qiagen) according to manufacturer’s instructions and treated with Turbo DNase (DNA-free kit, Ambion). DNA-free RNA was used to generate Cy3-labeled cDNA according to standard Nimblegen gene expression protocols. Reverse transcription was restricted to eukaryotic material by priming solely with oligo-dT primers. Labeled cDNA was hybridized to a Nimblegen 12-plex 135K human gene expression array. Expression data of Detroit 562 cells with and without *M. catarrhalis* was analyzed using Arraystar (DNASTAR) with Quantile RMA normalization. Genes that were not expressed in either condition, defined by a log_2_ SI of < 5, were omitted from the analysis. Statistical significance was assessed with a moderated t-test corrected with a Benjamini & Hochberg false discovery rate and considered significant at an adjusted *P* < 0.05 with a log_2_ fold-change of >1 or <-1. For the genes represented by multiple sequence identifiers after statistical testing, an average SI and corresponding fold-change was calculated.

### Tn-seq sequencing and microarray data

All microarray data have been deposited in NCBI Gene Expression Omnibus (GEO) database (www.ncbi.nlm.nih.gov/geo/) under GEO Series accession number GSE47870 for *M. catarrhalis* microarrays and GSE47711 for Detroit 562 microarrays. Tn-seq data can be found on the ESSENTIALS website (http://bamics2.cmbi.ru.nl/websoftware/essentials/links.html).

## Supporting Information

Table S1 **Genes identified by Tn-seq that affect adherence of** *M. catarrhalis* **BBH18 to A549** and **Detroit 562 cells.**
(XLSX)

Table S2 **Genes differentially expressed in Detroit 562-attached *M. catarrhalis* BBH18.**
(XLSX)

Table S3 **Detroit 562 genes induced in response to adherent *M. catarrhalis* BBH18.**
(XLSX)

Table S4 **Bacterial strains and plasmids used in this study.**
(DOCX)

Table S5 **Oligonucleotide primers used in this study.**
(DOCX)
