# Supplementary material for: Characterization of the Molecular Interplay between Moraxella catarrhalis and Human Respiratory Tract Epithelial Cells
Source: PLoS One. 2013 Aug 6;8(8):e72193. doi: 10.1371/journal.pone.0072193 (PMC3735583; doi:10.1371/journal.pone.0072193)
Supplement: Table S4 — Bacterial strains and plasmids used in this study. (DOCX) [file pone.0072193.s004.docx]

**Table S4**. **Bacterial strains and plasmids used in this study**

| Strain or plasmid | Relevant feature ^a^ | Reference |  |
| --- | --- | --- | --- |
| *Moraxella catarrhalis* |  |  | |
| BBH18 | Wild-type isolate | [1] | |
| BBH18 Δ*trmB* | *trmB* (MCR_0343) deletion mutant, Spec^R^ | This study | |
| BBH18 ΔMCR_0609 | BadM/Rrf2 family transcriptional regulator (MCR_0609) deletion mutant, Spec^R^ | This study | |
| BBH18 ΔMCR_0837 | putative phosphohistidine phosphatase (MCR_0837) deletion mutant, Spec^R^ | This study | |
| BBH18 Δ*aroA* | *aroA* (MCR_0888) deletion mutant, Spec^R^ | This study | |
| BBH18 Δ*encAB* | *ecnAB* (MCR_1029) deletion mutant, Spec^R^ | This study | |
| BBH18 Δ*lgt1* | *lgt1* (MCR_1095) deletion mutant, Spec^R^ | This study | |
| BBH18 ΔMCR_1483 | putative lipoprotein (MCR_1483) deletion mutant, Spec^R^ | This study | |
| BBH18 ΔMCR_1742 | outer membrane protein (MCR_1742) deletion mutant, Spec^R^ | This study | |
| *Plasmids* |  |  | |
| pR412T7 | Donor for Spec^R^ cassette | [2] | |
| pGSF8 | Donor for mariner transposon, containing Spec^R^ cassette and *mmeI* restriction sites | [3] | |

^a^ Spec^R^, spectinomycin resistance

**References**

1. Mollenkvist A, Nordstrom T, Hallden C, Christensen JJ, Forsgren A, et al. (2003) The *Moraxella catarrhalis* immunoglobulin D-binding protein MID has conserved sequences and is regulated by a mechanism corresponding to phase variation. J Bacteriol 185: 2285-2295.

2. Bijlsma JJ, Burghout P, Kloosterman TG, Bootsma HJ, de Jong A, et al. (2007) Development of genomic array footprinting for identification of conditionally essential genes in *Streptococcus pneumoniae*. Appl Environ Microbiol 73: 1514-1524.

3. Burghout P, Zomer AL, van der Gaast-de Jongh CE, Janssen-Megens EM, Francoijs KJ, et al. (2013) *Streptococcus pneumoniae* folate biosynthesis responds to environmental CO2-levels. J Bacteriol 195: 1573-1582.
