# Supplementary material for: Characterization of the Molecular Interplay between Moraxella catarrhalis and Human Respiratory Tract Epithelial Cells
Source: PLoS One. 2013 Aug 6;8(8):e72193. doi: 10.1371/journal.pone.0072193 (PMC3735583; doi:10.1371/journal.pone.0072193)
Supplement: Table S5 — Oligonucleotide primers used in this study. (DOCX) [file pone.0072193.s005.docx]

**Table S5**. **Oligonucleotide primers used in this study**

| Primer name | Target ^a^ | Sequence (5'- 3') |
| --- | --- | --- |
| Primers for generation of directed mutants | | |
| PBpR412_L | Spec^R^ cassette | GCCGCTCTAGAACTAGTGG |
| PBpR412_R | Spec^R^ cassette | GATACCCCTCGAATTGACGC |
| PBTnMr9 | Spec^R^ cassette control primer | CAATGGTTCAGATACGACGAC |
| *trmB-*L1-FW1 | *trmB* left flank | cctgccacacaatacaccac |
| *trmB-*L2-RV1 | *trmB* left flank | CCACTAGTTCTAGAGCGGCagacattttgggcatcttgg |
| *trmB-*R2-FW1 | *trmB* right flank | GCGTCAATTCGAGGGGTATCaatttgaaaagcgtggcatc |
| *trmB-*R1-RV1 | *trmB* right flank | gggtcaaggtggtgtaatgg |
| *trmB-*C-RV1 | *trmB* control primer | ttttacgaaactgctcaggtg |
| CvdGMCR_0609_L1 | MCR_0609 left flank | AGCACTCATGGTTCGTATGG |
| CvdGMCR_0609_L2 | MCR_0609 left flank | CCACTAGTTCTAGAGCGGCGTCCACGAGTTGTTAAACGC |
| CvdGMCR_0609_R2 | MCR_0609 right flank | GCGTCAATTCGAGGGGTATCCAAGTCAATCGCCTACATC |
| CvdGMCR_0609_R1 | MCR_0609 right flank | TCGGTAATGGTACCAAGCTC |
| CvdGMCR_0609_C | MCR_0609 control primer | ATCCAATAAGGCAGTGACGG |
| CvdGMCR_0837_L1 | MCR_0837 left flank | GCATTAGCCGAAATGAGTCG |
| CvdGMCR_0837_L2 | MCR_0837 left flank | CCACTAGTTCTAGAGCGGCAAAGTCGGTAAGATCTCGC |
| CvdGMCR_0837_R2 | MCR_0837 right flank | GCGTCAATTCGAGGGGTATCTTTGGCAACGACCCAAGATG |
| CvdGMCR_0837_R1 | MCR_0837 right flank | CTTATCCAAGTGAGCTACCC |
| CvdGMCR_0837_C | MCR_0837 control primer | TGTCTGCTGTACTTGCAGAC |
| CvdGMCR_0888-L1 | *aroA* (MCR_0888) left flank | CCAAATGTTGAACGAGTGGC |
| CvdGMCR_0888-L2 | *aroA* (MCR_0888) left flank | CCACTAGTTCTAGAGCGGCAGCCGATTACGCACAATTGC |
| CvdGMCR_0888-R2 | *aroA* (MCR_0888) right flank | GCGTCAATTCGAGGGGTATCTTGCCAATCGTGTTGGTCTG |
| CvdGMCR_0888-R1 | *aroA* (MCR_0888) right flank | CAATCTGAATGCCAACAGCG |
| CvdGMCR_0888-C | *aroA* (MCR_0888) control primer | GCATTGTGTACCGCTTGTAC |
| CvdGMCR_1029-L1 | *ecnAB* (MCR_1029) left flank | AGCAAGTGCCGATGAGATTG |
| CvdGMCR_1029-L2 | *ecnAB* (MCR_1029) left flank | CCACTAGTTCTAGAGCGGCAGTTTGTTGACTGCAGGCAG |
| CvdGMCR_1029-R2 | *ecnAB* (MCR_1029) right flank | GCGTCAATTCGAGGGGTATCAAGCACCTGAAGACCAACAG |
| CvdGMCR_1029-R1 | *ecnAB* (MCR_1029) right flank | TAATCAGCTGGAGTCTTGCC |
| CvdGMCR_1029-C | *ecnAB* (MCR_1029) control primer | GCTTGACTGGCTGAATCTTG |
| *lgt1*-L1-FW1 | *lgt1* (MCR_1095) left flank | taacgcataactgcctgtgg |
| *lgt1*-L2-RV1 | *lgt1* (MCR_1095) left flank | CCACTAGTTCTAGAGCGGCatatttggtcggtggaatgg |
| *lgt1*-R2-FW1 | *lgt1* (MCR_1095) right flank | GCGTCAATTCGAGGGGTATCgcagtcaaaccaaaagcaaag |
| *lgt1*-R1-RV2 | *lgt1* (MCR_1095) right flank | CAGGCACTGCAGGTTATGTC |
| *lgt1*-C-RV1 | *lgt1* (MCR_1095) control primer | aggtgcatgattggcattg |
| CvdGMCR_1483_L1 | MCR_1483 left flank | ATGCGAGCTTCAGGATTACC |
| CvdGMCR_1483_L2 | MCR_1483 left flank | CCACTAGTTCTAGAGCGGCGACCACAAGCTGAAAGCAAAC |
| CvdGMCR_1483_R1 | MCR_1483 right flank | GCGTCAATTCGAGGGGTATCACCCATCAGCGATGATATG |
| CvdGMCR_1483_R2 | MCR_1483 right flank | TTGGCATCGGCAACTTGAAG |
| CvdGMCR_1483_C | MCR_1483 control primer | GCGTACTTTCATCGGGTAG |
| CvdGMCR_1742_L1 | MCR_1742 left flank | TTCGTAAGCTCATGCAACCC |
| CvdGMCR_1742_L2 | MCR_1742 left flank | CCACTAGTTCTAGAGCGGCTAGCGATTAGTGCAGATGCG |
| CvdGMCR_1742_R1 | MCR_1742 right flank | GCGTCAATTCGAGGGGTATCTGCAACTGAAGCAACCGAAC |
| CvdGMCR_1742_R2 | MCR_1742 right flank | TCATCTTCGGTGATATCGCC |
| CvdGMCR_1742_C | MCR_1742 control primer | GAGCAAGCAGTTAGTGCTAG |

^a^ Spec^R^, spectinomycin resistance
